# *RIPK1* polymorphisms alter the susceptibility to cervical Cancer among the Uyghur population in China

**DOI:** 10.1186/s12885-020-06779-4

**Published:** 2020-04-09

**Authors:** Zulipiyamu Tuoheti, Lili Han, Sulaiya Husaiyin, Xiaoxi Liu, Chunhua Ma, Mayinuer Niyazi

**Affiliations:** 1 Department of Gynecology, People’s Hospital of Xinjiang Uygur Autonomous Region, No 91 Tianqi Road, Urumqi, Xinjiang, 830001 China; 2 Key Laboratory of Resource Biology and Biotechnology in Western China, (Northwest University), Ministry of Education, Xi’an, Shaanxi 710069 P. R. China

**Keywords:** *RIPK1*, Cervical cancer, Uyghur, Case-control study

## Abstract

**Background:**

*RIPK1* (receptor-interacting protein kinase-1) plays a role in cancer development, whereas no clear studies focused on the cervical cancer. The objective of this study was to evaluate the relationship between *RIPK1* polymorphisms and cervical cancer risk among the Uyghur population.

**Methods:**

We performed a case-control study including 342 cervical cancer patients and 498 age-matched healthy controls. Four *RIPK1* genetic variants (rs6907943, rs2077681, rs9503400 and rs17548629) were genotyped with Agena MassARRAY platform. The associations between *RIPK1* polymorphisms and cervical cancer risk were assessed under Binary logistic regression models. False discovery rate (FDR) was used to improve the results reliability.

**Results:**

The results showed rs2077681 was significantly associated with cervical cancer risk under various genetic models (codominant: OR = 3.14, 95% CI = 1.40–7.07, *p* = 0.006, FDR-*p* = 0.018; recessive: OR = 3.20, 95% CI = 1.43–7.16, *p* = 0.005, FDR-0.018). The stratified analysis indicated that the relationships of rs6907946, rs9503400 and rs17548629 with cervical cancer risk were statistically significant in the subgroup of clinical stage (*p* < 0.05).

**Conclusion:**

Our findings demonstrated that *RIPK1* polymorphisms were associated with cervical cancer susceptibility among the Uyghur population in China, and *RIPK1* polymorphisms might be involved in the development of cervical cancer.

## Background

Cervical cancer is the fourth most common cancer in women worldwide, and it remains the leading cause of cancer death for women [1]. It was reported that there were approximately 500,000 new cases of cervical cancer annually and most cases occurred in developing countries [2]. In China, the incidence and mortality of cervical cancer continue to increase, especially among women living in rural [3]. The Uyghur, one of minorities in China, has higher morbidity and mortality of cervical cancer than other ethnic groups [4]. However, the mechanism underlying cervical cancer remains unclear. Recently, accumulating evidences indicate that genetic factors play a vital role in the development of cervical cancer. To unveil the genetic susceptibility of cervical cancer, it is important to identify genetic markers that affect the cervical cancer development.

*Receptor-interacting protein kinase-1* (*RIPK1*) gene encodes a member of the receptor- interacting protein family of serine/threonine protein kinases. *RIPK1* mediates the upstream of NF-κB signaling and plays a crucial role in inflammation and cell death [5, 6]. Moreover, *RIPK1* was identified as a key effector molecule of necroptosis [7]. Number of evidences suggested that necroptosis might take part in the regulation of cancer by pro-inflammatory cytokine production and anti-tumor immune response [8]. It has been reported that *RIPK1* is implicated in some diseases, such as chronic periodontitis [9], liver diseases, and cancers [10]. In mouse models of liver injury, several studies highlighted the importance of *RIPK1* in regulating hepatocyte apoptosis through distinct kinase functions [11]. Additionally, genetic variants of *RIPK1* may alter the ability of the gene to bind substance, activate transcription and induce apoptosis [12, 13]. Evidences have suggested *RIPK1* polymorphisms could be a possible biomarker in colorectal cancer [12]. For example, Chae et al. showed *RIPK1* polymorphism was associated with increased risk of colorectal cancer and poorer prognosis of colorectal cancer patients [14]. Nonetheless, the role of *RIPK1* polymorphisms has not been confirmed in cervical cancer.

Therefore, to further explore the role of *RIPK1* polymorphisms in cervical cancer, we conducted a case-control study to assess the impact of *RIPK1* polymorphisms in cervical cancer susceptibility among the Uyghur population in China.

## Methods

### Study population

A total of 342 newly diagnosed and histologically confirmed cervical cancer patients were consecutively recruited from People’s Hospital of Xinjiang Uygur Autonomous Region. During the same period, we randomly chosen healthy controls who underwent the health examination in People’s Hospital of Xinjiang Uygur Autonomous Region. The healthy controls were matched with patients in age. All participators were Uyghur population living in Xin’jiang Province of China. Individuals with cancer history, viral infection, diabetes or cardiovascular diseases were excluded in this study. Written informed consent was obtained from each participant before the sample collection. The study was approved by the Committee for Ethical Affairs of People’s Hospital of Xinjiang Uygur Autonomous Region, and study was performed according to the declaration of Helsinki.

### SNP selection and genotyping

Candidate SNPs of *RIPK1* gene were selected from previous studies, and preliminary analysis of *RIPK1* polymorphism was done using the 1000 Genomes database with minor allele frequency (MAF) larger than 0.05 [15]. Genomic DNA was extracted from peripheral blood samples via a blood DNA kit (GoldMag Co. Ltd., Xi′an, China), and quantified with Nanodrop 2000 (Thermo Scientific, Waltham, Massachusetts, USA). MassARRAY Assay Design 3.0 software was applied for primers design (Table 1). The SNP genotyping was performed using MassARRAY iPLEX platform (Agena Bioscience, San Diego, CA, USA) according to the manufacturer’s instructions [16]. Finally, the genotyping results were managed and outputted by Agena Bioscience TYPER version 4.0 software.

**Table 1:** Primer sequences used for this study

SNP	First-PCRP	Second-PCRP	UEP_DIR	UEP_SEQ
rs6907943	ACGTTGGATGACCAGGTGTTGGAGTTCAGC	ACGTTGGATGGGTGTTTGTTTGCAGCTCGT	F	tgtgTGCAGCTCGTTAGCAT
rs2077681	ACGTTGGATGGTGAATTTAACTGCACTGGG	ACGTTGGATGAACCTCGAGGACATCATGCC	R	ggggtACATCATGCCAAGTGGA
rs9503400	ACGTTGGATGAGTAAGTGCTCAGTAAACGG	ACGTTGGATGTGCTCAAGGCTGTCTAGGTG	R	ggagGGCTGTCTAGGTGTTCTTTG
rs17548629	ACGTTGGATGTCAACAGTATCAGCCCTGAG	ACGTTGGATGTGGCATTCTGGTACCTTCAC	F	ccccTCACCCAGCCTGAGTG

*SNP* Sing nucleotide polymorphism

### Statistical analysis

All data were analyzed using the SPSS 22.0 software (IBM®, Armonk, New York, USA). We used Student’s *t*-test for continuous variables to evaluate the difference of characteristics between two groups [17]. The Hardy-Weinberg equilibrium (HWE) values were calculated for the SNPs in the healthy control using Fisher exact test. The differences between cases and controls in the frequency of the alleles and genotypes were evaluated by Chi-square analysis. The functions of candidate SNPs were predicted by HaploReg v4.1. The association between genetic variants and cervical cancer risk was assessed by odd ratios (OR) and 95% confidence interval (CI) from Binary logistic regression analyses in genetic models [18]. Age was regarded as a covariate in the logistic regression analysis. Then, we used the Haploview software (version 4.2) and the PLINK software to analyze linkage disequilibrium (LD) and haplotype. All tests were two-sided and the statistical significance was set at *p* < 0.05. False discovery rate (FDR) was used to correct *p* values.

## Results

Characteristics of 840 subjects were summarized in Table 2. A total of 342 cervical cancer patients and 498 healthy controls were enrolled with mean ages of 43.27 ± 11.78 and 43.46 ± 13.03 years, respectively. There was no significant difference in age distribution between cases and controls. According the 2009 FIGO staging system, we divided 342 cervical cancer cases into different clinical stages, 132 cases (39%) were in stage I and II, 80 cases (23%) were in stage III and IV.

**Table 2:** Characteristics of the cervical cancer patients and healthy controls in this study

Characteristics	Cervical cancer cases (***N*** = 342)	Healthy controls (***N*** = 498)	***P*** Value
Age (year, mean ± SD)	43.27 ± 11.78	43.46 ± 13.03	0.832
> 43	176 (51%)	263 (53%)	
≤ 43	166 (49%)	235 (47%)	
Stage (%)			
I II	132 (39%)		
III IV	80 (23%)		
Absence	130 (38%)		

*SD* Standard deviation

Detailed information of *RIPK1* polymorphisms, including chromosome, position, allele, genotype and allele distribution, MAF and HWE *p* value were listed in Table 3. The distribution frequencies of four SNPs were in HWE (*p* > 0.05). HaploReg showed that *RIPK1* polymorphisms were related to the regulations of Enhancer histone marks, Motifs changed, and Selected eQTL hits. The relationship between *RIPK1* polymorphisms and cervical cancer risk was shown in Table 4. Compared with the healthy controls, the individuals who carried CC genotype of rs2077681 had higher risk of cervical cancer (codominant: OR = 3.14, 95% CI = 1.40–7.07, *p* = 0.006; recessive: OR = 3.20, 95% CI = 1.43–7.16, *p* = 0.005). FDR analysis verified the reliability of these results (codominant: FDR*-p* = 0.018; recessive: FDR*-p* = 0.018). There were no significant associations between other SNPs and cervical cancer risk in this study (*p* > 0.05).

**Table 3:** Detail information of the *RIPK1* gene polymorphisms

SNP	Chromosome	BP	Alleles	Group	Genotype			Allele		MAF	HWE ***p***	HaploReg
rs6907943	6	3,078,032	A/C		CC	CA	AA	C	A			Enhancer histone marks, Motifs changed, Selected eQTL hits
				Control	13 (2.62%)	129 (25.96%)	355 (71.42%)	155 (15.59%)	839 (84.41%)	0.156	0.734	
				Case	15 (4.40%)	77 (22.58%)	249 (73.02%)	107 (15.69%)	575 (84.31%)	0.157		
rs2077681	6	3,085,866	A/G		CC	CT	TT	C	T			Motifs changed, Selected eQTL hits
				Control	9 (1.82%)	140 (28.28%)	346 (69.90%)	158 (15.96%)	832 (84.04%)	0.160	0.313	
				Case	19 (5.57%)	89 (26.10%)	233 (68.33%)	127 (18.62%)	555 (81.38%)	0.186		
rs9503400	6	3,108,673	A/G		AA	AG	GG	A	G			Enhancer histone marks, Motifs changed
				Control	1 (0.20%)	47 (9.44%)	450 (90.36%)	49 (4.92%)	947 (95.08%)	0.051	1.000	
				Case	3 (0.88%)	39 (11.40%)	300 (87.72%)	45 (6.58%)	639 (93.42%)	0.066		
rs17548629	6	3,114,223	C/T		TT	TC	CC	T	C			Motifs changed
				Control	9 (1.81%)	122 (24.50%)	367 (73.69%)	140 (14.06%)	856 (85.94%)	0.141	0.855	
				Case	10 (2.92%)	72 (21.05%)	260 (76.03%)	92 (13.45%)	592 (86.55%)	0.134		

*SNP* Sing nucleotide polymorphism, *MAF* Minor allele frequency, *HWE* Hardy weinberg equilibrium

**Table 4:** The association of *RIPK1* gene polymorphisms with cervical cancer susceptibility in Uygur population

SNP	Model	Allele/Genotype	Frequency (Control/Case)	OR (95%CI)	***P*** Value	FDR***-p*** Value
rs6907943	Allele	C	15.59%/15.69%	1.01 (0.77–1.32)	0.958	0.958
		A	84.41%/84.31%	1.00		
	Codominant	CC	2.62%/4.40%	1.65 (0.77–3.52)	0.199	0.597
		CA	25.96%/22.58%	0.85 (0.61–1.18)	0.333	0.666
		AA	71.42%/73.02%	1.00		
	Dominant	CC-CA	28.58%/26.98%	0.92 (0.68–1.26)	0.616	0.924
		AA	71.42%/73.02%	1.00		
	Recessive	CC	2.62%/4.40%	1.72 (0.81–3.65)	0.162	0.597
		CA-AA	97.38%/95.60%	1.00		
	Log-additive			1.01 (0.78–1.31)	0.956	0.958
rs2077681	Allele	C	15.96%/18.62%	1.21 (0.93–1.56)	0.155	0.245
		T	84.04%/81.38%	1.00		
	Codominant	CC	1.82%/5.57%	3.14 (1.40–7.07)	**0.006**	**0.018**
		CT	28.28%/26.10%	0.94 (0.69–1.29)	0.711	0.711
		TT	69.90%/68.33%	1.00		
	Dominant	CC-CT	30.10%/31.67%	1.08 (0.80–1.45)	0.634	0.711
		TT	69.90%/68.33%	1.00		
	Recessive	CC	1.82%/5.57%	3.20 (1.43–7.16)	**0.005**	**0.018**
		CT-TT	98.18%/94.43%	1.00		
	Log-additive			1.20 (0.93–1.54)	0.163	0.245
rs9503400	Allele	A	4.92%/6.58%	1.36 (0.90–2.07)	0.146	0.271
		G	95.08%/93.42%	1.00		
	Codominant	AA	0.20%/0.88%	4.46 (0.46–43.25)	0.197	0.271
		GA	9.44%/11.40%	1.25 (0.79–1.95)	0.339	0.339
		GG	90.36%/87.72%	1.00		
	Dominant	AA-GA	9.64%/12.28%	1.31 (0.85–2.04)	0.226	0.271
		GG	90.36%/87.72%	1.00		
	Recessive	AA	0.20%/0.88%	4.36 (0.45–42.26)	0.203	0.271
		GA-GG	99.8%/99.12%	1.00		
	Log-additive			1.35 (0.89–2.03)	0.155	0.271
rs17548629	Allele	T	14.06%/13.45%	0.95 (0.72–1.26)	0.724	0.724
		C	85.94%/86.55%	1.00		
	Codominant	TT	1.81%/2.92%	1.57 (0.63–3.92)	0.334	0.663
		TC	24.50%/21.05%	0.83 (0.60–1.16)	0.277	0.663
		CC	73.69%/76.03%	1.00		
	Dominant	TT-TC	26.31%/23.97%	0.88 (0.64–1.21)	0.442	0.663
		CC	73.69%/76.03%	1.00		
	Recessive	TT	1.81%/2.92%	1.64 (0.66–4.07)	0.289	0.663
		TC-CC	98.19%/97.08%	1.00		
	Log-additive			0.95 (0.72–1.26)	0.724	0.724

*SNP* Sing nucleotide polymorphism, *OR* Odds ratios, *CI* Confidence intervals, *FDR* False discovery ratel

Bold italics indicates the SNP with statistical significance (*p* < 0.05)

We then performed stratification analysis on the association of *RIPK1* polymorphisms with cervical cancer susceptibility (Table 5). In the subgroup of age >  43 years, individuals carrying the genotype CC in rs2077681 (codominant: OR = 3.38, 95% CI = 1.15–9.99, *p* = 0.027; recessive: OR = 3.46, 95% CI = 1.18–10.15, *p* = 0.024) and TT in rs17548629 (codominant: OR = 3.99, 95% CI = 1.04–15.32, *p* = 0.044; recessive: OR = 4.09, 95% CI = 1.07–15.63, *p* = 0.040) were more likely to suffer from cervical cancer, whereas FDR analysis showed the strong linkages of rs2077681 and rs17548629 with cervical cancer may not be reliable (FDR- *p* > 0.05). Moreover, we found that rs6907943 and rs17548629 exerted protective roles in higher grade cervical cancer among Uyghur population (rs6907943, allele: OR = 0.47, 95% CI = 0.24–0.90, *p* = 0.021; dominant: OR = 0.46, 95% CI = 0.23–0.93, *p* = 0.031; log-additive: OR = 0.46, 95% CI = 0.23–0.89, *p* = 0.022; rs17548629, allele: OR = 0.41, 95% CI = 0.21–0.83, *p* = 0.011; dominant: OR = 0.44, 95% CI = 0.21–0.93, *p* = 0.031; log-additive: OR = 0.43, 95% CI = 0.22–0.87, *p* = 0.018). However, allele (OR = 3.42, 95% CI = 1.55–7.56, *p* = 0.001), dominant (OR = 3.57, 95% CI = 1.55–8.22, *p* = 0.003) and log-additive (OR = 3.53, 95% CI = 1.56–7.98, *p* = 0.002) models revealed the remarkable associations of rs9503400 and increased risk of stage III/IV cervical cancer. Haplotype analysis did not show blocks in *RIPK1* polymorphisms (Supplemental Figure 1), and no associations with risk of cervical cancer.

**Table 5:** Association of *RIPK1* gene polymorphisms with cervical cancer susceptibility after stratifying by age and stage

SNP	Model	Age (Cases Vs. Controls)						Stage (III/IV Vs. I/II)		
		> 43 (176 Vs. 263)			≤ 43 (166 Vs. 235)			80 Vs. 132		
		OR (95%CI)	***p*** Value	FDR***-p*** Value	OR (95%CI)	***p*** Value	FDR***-p*** Value	OR (95%CI)	***p*** Value	FDR***-p*** Value
rs6907943	Allele (15.91–14.83%/84.09–85.17%)	1.09 (0.75–1.58)	0.663	0.796	0.93 (0.63–1.37)	0.705	0.705	0.47 (0.24–0.90)	**0.021**	**0.041**
	Codominant	1.91 (0.69–5.26)	0.211	0.633	1.35 (0.42–4.29)	0.614	0.705	–	–	–
		0.89 (0.56–1.42)	0.629	0.796	0.80 (0.50–1.27)	0.335	0.705	0.50 (0.25–1.02)	0.056	0.056
	Dominant	0.99 (0.65–1.53)	0.979	0.979	0.84 (0.54–1.31)	0.449	0.705	0.46 (0.23–0.93)	**0.031**	**0.041**
	Recessive	1.96 (0.72–5.37)	0.190	0.633	1.43 (0.45–4.53)	0.542	0.705	–	**–**	**–**
	Log-additive	1.08 (0.76–1.55)	0.656	0.796	0.92 (0.63–1.34)	0.650	0.705	0.46 (0.23–0.89)	**0.022**	**0.041**
rs2077681	Allele (18.57–15.46%/81.43–84.54%)	1.25 (0.87–1.79)	0.227	0.387	1.16 (0.80–1.68)	0.430	0.698	0.69 (0.40–1.20)	0.187	0.306
	Codominant	3.38 (1.15–9.99)	**0.027**	0.081	2.83 (0.83–9.66)	0.096	0.288	1.39 (0.33–5.77)	0.652	0.652
		0.92 (0.59–1.43)	0.708	0.717	0.94 (0.60–1.47)	0.779	0.851	0.47 (0.24–0.94)	**0.034**	0.204
	Dominant	1.08 (0.71–1.64)	0.717	0.717	1.04 (0.68–1.6)	0.851	0.851	0.55 (0.29–1.05)	0.070	0.210
	Recessive	3.46 (1.18–10.15)	**0.024**	0.081	2.89 (0.85–9.77)	0.088	0.288	1.67 (0.41–6.89)	0.478	0.574
	Log-additive	1.22 (0.86–1.73)	0.258	0.387	1.15 (0.79–1.66)	0.465	0.698	0.71 (0.42–1.21)	0.204	0.306
rs9503400	Allele (6.25–4.94%/93.75–95.06%)	1.28 (0.71–2.30)	0.404	0.404	1.45 (0.80–2.63)	0.222	0.347	3.42 (1.55–7.56)	**0.001**	**0.004**
	Codominant	–	–	–	4.41 (0.45–42.80)	0.201	0.347	–	–	–
		–	–	–	1.21 (0.61–2.37)	0.585	0.585	3.37 (1.45–7.82)	0.005	0.005
	Dominant	1.30 (0.71–2.38)	0.392	0.404	1.36 (0.71–2.58)	0.355	0.426	3.57 (1.55–8.22)	**0.003**	**0.004**
	Recessive	–	–	–	4.33 (0.45–41.98)	0.207	0.347	–	**–**	**–**
	Log-additive	1.30 (0.71–2.38)	0.392	0.404	1.41 (0.80–2.49)	0.231	0.347	3.53 (1.56–7.98)	**0.002**	**0.004**
rs17548629	Allele (14.49–12.36%/85.51–87.64%)	1.20 (0.81–1.78)	0.361	0.579	0.74 (0.49–1.12)	0.153	0.355	0.41 (0.21–0.83)	**0.011**	**0.036**
	Codominant	3.99 (1.04–15.32)	**0.044**	0.132	0.44 (0.09–2.20)	0.315	0.355	–	–	–
		0.89 (0.56–1.44)	0.645	0.774	0.76 (0.48–1.22)	0.256	0.355	0.52 (0.24–1.10)	0.086	0.086
	Dominant	1.05 (0.67–1.63)	0.846	0.846	0.73 (0.47–1.16)	0.185	0.355	0.44 (0.21–0.93)	**0.031**	**0.041**
	Recessive	4.09 (1.07–15.63)	**0.040**	0.132	0.47 (0.09–2.34)	0.355	0.355	–	**–**	**–**
	Log-additive	1.19 (0.81–1.74)	0.386	0.579	0.74 (0.49–1.11)	0.147	0.355	0.43 (0.22–0.87)	**0.018**	**0.036**

*SNP* Sing nucleotide polymorphism, *OR* Odds ratios, *CI* Confidence intervals, *FDR* False discovery rate

Bold italics indicates the SNP with statistical significance (*p* < 0.05)

## Discussion

In the present study, we found strong linkages between *RIPK1* polymorphisms and cervical cancer susceptibility. To our knowledge, it is the first study to provide the evidence that *RIPK1* polymorphisms are associated with cervical cancer risk among the Uygur population in China.

*RIPK1*, also known as *RIP1*, is a main adaptor kinase in several signaling pathways inducing tumor cell apoptosis by activing NF-κB [19, 20]. The overexpression of *RIPK1* was associated with a poor prognosis for brain tumors based on altering the apoptosis [21]. Besides that, smac mimetics are considered as potential cancer therapeutics. It has been demonstrated that *RIPK1* was involved in SM-induced cell death in breast and lung cancer cell [22, 23]. Chae et al. found that *RIPK1* polymorphism is an indicator of hepatic injury and a promising prognostic biomarker for cancer development, whereas *RIPK1* polymorphisms were not associated with rectal cancer [12]. This difference may be attributed to the biologic differences of *RIPK1* polymorphisms. Genetic mutations are dependent on cancer site, etiology of cancer, study population and environmental factors. Moreover, cancer is a heterogeneous disease in terms of risk factor, tumor features and somatic alterations [24, 25]. In our study, *RIPK1* rs2077681was remarkablely related to cervical cancer risk after adjustment. Specially, it was observed that age and stage of cervical cancer could affect the relationship between four *RIPK1* polymorphisms and cervical cancer among the Uyghur population.

Many risks are related to the incidence of cervical cancer, including individuals’ characteristics, oncogenic HPV infections, smoking habits and other disease [26]. As we all know, aging is an obvious risk for the development of cancer [27]. Hence, we explored the association between *RIPK1* polymorphisms and cervical cancer susceptibility stratified by age. We did not observe significant associations between *RIPK1* polymorphisms and risk of cervical cancer. It suggests that age is not an important factor for the association of *RIPK1* polymorphisms and cervical cancer. The expression or mutation of *RIPK1* polymorphisms with aging would not be deleterious for cervical cancer. Furthermore, we found the genetic variants in *RIPK1* contribute to different clinical outcomes among cervical cancer patients, which suggests the necessity of the study on genetic susceptibility. *RIPK1* rs6907943 and rs17548629 were protective factors for the higher-grade (III, IV) cervical cancer among the Uyghur population by stratification analysis. However, rs9503400 increased the risk for cervical cancer patients during stage III and IV. It may be attributed to the regulation of *RIPK1* polymorphisms on Enhancer histone marks and Motifs changed. These results indicated the impact of candidate SNPs on cervical cancer risk, providing evidences for prevention, diagnosis and personalized treatment of cervical cancer.

There are several limitations in the present study. First, the limited sample size, more samples are needed to validate our findings. Second, we did not analyze the impact of other risk factors on the cervical cancer susceptibility due to the lack of information on participates. Third, some patients were lacking clinical stage information, it may influence the stratified analysis results. Finally, although we identified the close associations between *RIPK1* polymorphisms and cervical cancer risk, the underlying mechanism is still unclear. Further studies are required to perfect our study.

## Conclusion

In conclusion, our study reveals that *RIPK1* polymorphisms alter the susceptibility to cervical cancer among the Uyghur population, and it suggests that *RIPK1* polymorphisms exert significant roles in cervical cancer development. Large-scale studies of different ethnic groups are required to validate the genetic association and functional studies are also needed to unveil the underlying mechanism of the *RIPK1* polymorphisms on cervical carcinogenesis.

## Supplementary information

**Additional file 1: Supplemental Figure S1.** Haplotype block map for the SNPs of *RIPK1.* The LD between two SNPs is standardized by D′.

## Data Availability

The datasets used and/or analyzed during the current study available from the corresponding author on reasonable request.

## Abbreviations

*RIPK1*: Receptor-interacting protein kinase-1)
MAF: Minor allele frequency
HWE: Hardy-Weinberg equilibrium
OR: Odd ratios
CI: Confidence interval
LD: Linkage disequilibrium
FDR: False discovery rate
