# Supplementary figures and images for: RIPK1 polymorphisms alter the susceptibility to cervical Cancer among the Uyghur population in China
Source: BMC Cancer. 2020 Apr 9;20:299. doi: 10.1186/s12885-020-06779-4 (PMC7146988; doi:10.1186/s12885-020-06779-4)

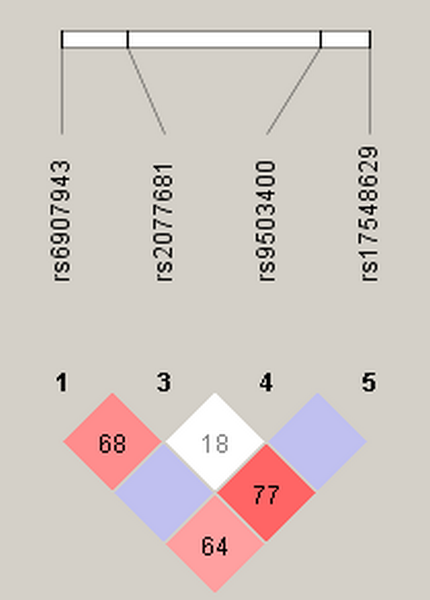

Supplement: Supplementary file 1 — Additional file 1: Supplemental Figure S1. Haplotype block map for the SNPs of RIPK1. The LD between two SNPs is standardized by D′. [file 12885_2020_6779_MOESM1_ESM.tif]
